# Inducible intracellular membranes: molecular aspects and emerging applications

**DOI:** 10.1186/s12934-020-01433-x

**Published:** 2020-09-04

**Authors:** Jorge Royes, Valérie Biou, Nathalie Dautin, Christophe Tribet, Bruno Miroux

**Affiliations:** 1 Laboratoire de Biologie Physico-Chimique des Protéines Membranaires, Université de Paris, LBPC-PM, CNRS, UMR7099, 75005 Paris, France; 2 Institut de Biologie Physico-Chimique, Fondation Edmond de Rothschild pour le Développement de la Recherche Scientifique, 75005 Paris, France; 3 Département de Chimie, École Normale Supérieure, PASTEUR, PSL University, CNRS, Sorbonne Université, 24 Rue Lhomond, 75005 Paris, France

**Keywords:** Membrane remodeling, Membrane biosynthesis, Membrane curvature, Phospholipids, Inner membrane, Lipid biosynthesis

## Abstract

Membrane remodeling and phospholipid biosynthesis are normally tightly regulated to maintain the shape and function of cells. Indeed, different physiological mechanisms ensure a precise coordination between de novo phospholipid biosynthesis and modulation of membrane morphology. Interestingly, the overproduction of certain membrane proteins hijack these regulation networks, leading to the formation of impressive intracellular membrane structures in both prokaryotic and eukaryotic cells. The proteins triggering an abnormal accumulation of membrane structures inside the cells (or membrane proliferation) share two major common features: (1) they promote the formation of highly curved membrane domains and (2) they lead to an enrichment in anionic, cone-shaped phospholipids (cardiolipin or phosphatidic acid) in the newly formed membranes. Taking into account the available examples of membrane proliferation upon protein overproduction, together with the latest biochemical, biophysical and structural data, we explore the relationship between protein synthesis and membrane biogenesis. We propose a mechanism for the formation of these non-physiological intracellular membranes that shares similarities with natural inner membrane structures found in α-proteobacteria, mitochondria and some viruses-infected cells, pointing towards a conserved feature through evolution. We hope that the information discussed in this review will give a better grasp of the biophysical mechanisms behind physiological and induced intracellular membrane proliferation, and inspire new applications, either for academia (high-yield membrane protein production and nanovesicle production) or industry (biofuel production and vaccine preparation).

## Background

Biological membranes are complex, two-dimensional structured assemblies of phospholipids containing a high density of proteins and carbohydrates. The possibility of controlling the production and organization of biological membranes is still an open question and it has several implications for biotechnology. For example, the increased phospholipid amount due to membrane expansion is useful in the field of biofuel production by fermentation. Modification of metabolic pathways aiming at diverting carbon fluxes towards the desired target compound has been tried and is far from being straightforward [1, 2]. In this context, the overexpression of a protein triggering membrane proliferation represents a simple, alternative strategy to redirect lipid metabolism and enhance biofuel production yield. In the same line, production of the P9 and P12 phage φ6 viral proteins have been proposed to increase the yield of useful hydrophobic active principles [3].

For structural biologists, membrane protein production still represents a major technological challenge [4]. Despite the emergence of eukaryotic expression systems, prokaryotic expression systems are the most popular vehicle for membrane protein production [5]. Historically, genetically modified strains have been developed to enhance membrane protein production [6–8]. Recently, a novel strategy has arisen for membrane protein production both in prokaryotes and eukaryotes, relying on tuning the cell membrane phospholipid composition to accommodate higher amounts of recombinant membrane proteins [9, 10]. Alternatively, some of the proteins triggering internal membrane proliferation have been proposed as a fusion partner to membrane proteins to trigger membrane expansion and increase the yield of membrane protein production [11]. The next generation of membrane protein production platforms may combine those three strategies: genetic regulation of protein expression, modulation of phospholipid composition, and membrane expansion triggered by protein overproduction.

Membrane production platforms could also find applications in nanotechnology and nanomedicine. Almost all cell types secrete nano- and micro-sized vesicles used for intercellular communication [12, 13]. Granting control over the production and composition of those vesicles hold great promises in nanotechnology and nanomedicine [14, 15]. In this regard, the protein-induced intracellular membrane proliferation has been suggested as a new route to increase production yield of microvesicles for antiviral or tumoral treatments, or as contrast agents in bioimaging [16–19]. Membrane proliferation upon overproduction of the b subunit of F_o_F_1_-ATP synthase has been recently used to prepare proteoliposomes [20]. This method represents an attractive alternative to in vitro proteoliposomes reconstitution, alleviating several steps of protein extraction, purification and reconstitution in liposomes. In the same line, the preparation of bacteria-derived lipid vesicles presenting antigenic proteins from pathogens on their surface have been used for vaccine preparation [21, 22]. The production of chimera proteins containing a membrane-proliferation domain and an adequate antigen could dramatically improve vaccine safety and mass production.

Here, we will critically review and rationalize the available knowledge gained from studying membrane remodeling in physiological context to find the basic physico-chemical principles governing membrane production that can be applied to *inducible* non-physiological membrane rearrangements. We hope that the mechanistic principles proposed in this review can help to harness this phenomenon in the design of new biotechnological applications.

### Influence of membrane curvature in membrane remodeling

From a biological standpoint, membranes are lipidic films that define the boundaries of cells and organelles. They constitute permeability barriers and major sites of exchange between the interior and exterior of these cells and compartments. Thus, they are essential for compartmentalizing the biochemical reactions that sustain life. Membranes are composed of lipids arranged as bilayers, together with proteins that can be either inserted in the lipid layer or peripherally associated to it. Membrane organization, as well as lipid and protein constituents, vary between organisms (eukaryotic cells, bacteria, virus), but also among species of certain organism. Furthermore, membrane composition changes in response to various signals or environmental conditions resulting in three-dimensional rearrangements, or membrane remodeling events. These spatial rearrangements occur in all life forms; however, the exact mechanisms underlying these events have mainly been deciphered in eukaryotic cells and are starting to be understood in prokaryotes.

**Fig. 1:**
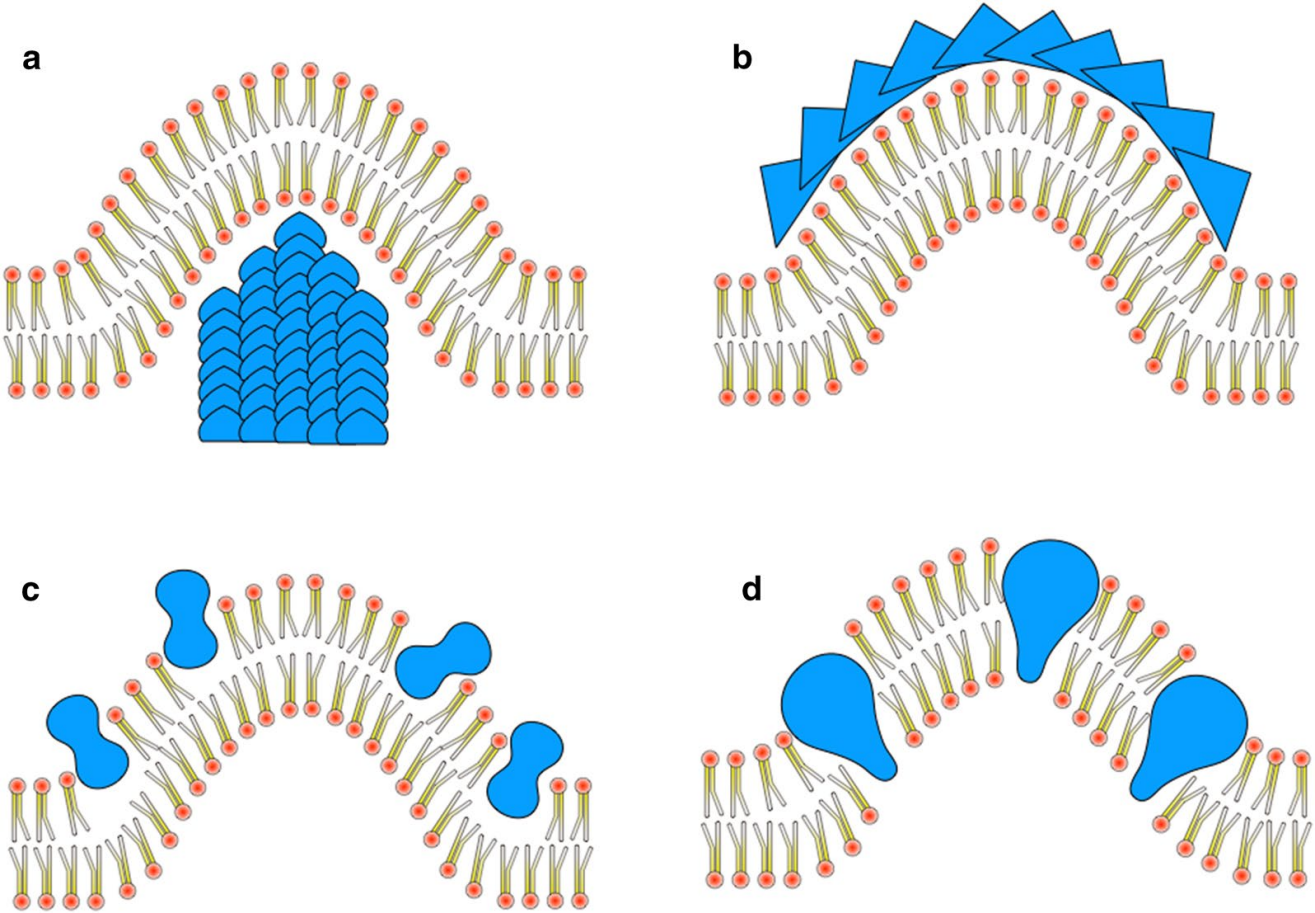
Different mechanisms of membrane deformation **a** push force by molecular motors, **b** protein supramolecular scaffolding, **c** asymmetric interaction with one leaflet of the membrane and **d** insertion of wedged-shaped proteins.

Eukaryotic cells possess various essential intracellular organelles (endoplasmic reticulum (ER), Golgi apparatus, endosome, mitochondria...), which differ in morphology, function and structure but are all bounded by bilayers with increased curvature compared to the cell membrane. In addition, multiple membrane remodeling events have to be coordinated to carry out physiological processes such as vesicular trafficking, endocytosis or exocytosis. These processes, which have been studied for decades, depend on a large array of proteins, either cytoskeletal (actin, tubulin) or directly implicated in membrane curvature and remodeling, such as clathrin, dynamins or BAR (Bin-Amphiphysin-Rvs) or ENTH (Epsin NH2-Terminal Homology) domain-containing proteins. For a long time, these complex membrane remodeling processes were thought to be an exclusive features of eukaryotic cells. Recently, it has been demonstrated that prokaryotic cells also undergo multiple membrane remodeling processes which are similarly controlled by specific proteins, analogous to the ones found in eukaryotic cells [23]. Regardless of the different protein complexes involved for each organism, from a biophysical perspective, four basic molecular mechanisms have been described to remodel biological membrane, modifying their curvature (Fig. 1) [24]:

- Pushing or pulling the membrane using molecular motors.
- Bending along a rigid supramolecular protein scaffold.
- Asymmetric interaction of proteins with only one leaflet of the lipid bilayer.
- Insertion of wedge-shaped proteins into the membrane.

Those biophysical mechanisms are ubiquitous and involved in multiple physiological processes.

#### Examples of proteins modifying membrane-curvature

Proteins with ENTH domains in eukaryotes insert a wedged-shaped N-terminal amphipathic helix into the membrane, leading to curvature and deformation of the membrane (Fig. 1a). ENTH-containing proteins are also involved in clathrin mediated budding by regulating and promoting the scaffolding of clathrin on membrane (Fig. 1b). However, for the moment proteins with ENTH domains have only been observed in eukaryotes.

Another example are BAR domains, which form banana-shaped dimers that can bind membrane with curvature but also induce and stabilize membrane curvature via helical oligomerization and scaffolding [25]. BAR domain interaction also leads to phosphoinositides lipid clustering and formation of stable microdomains [26]. BAR-domain proteins functions are multiple: they regulate actin polymerization by interacting and recruiting actin assembly factors at the membrane, they cooperate with dynamin and clathrin to mediate membrane fission during endocytosis and are involved in the formation of filopodia and lamellipodia [25]. Only 2 BAR-containing proteins have been described so far in bacteria, BdpA from *Shewanella oneidensis* is involved in the biogenesis of outer membrane extensions and trigger the formation of such extensions when expressed in a heterologous host (*E. coli*) [27]. MamY is a BAR-domain protein implicated in magnetosome formation [28].

The Dynamin superfamily of proteins includes some of the best studied membrane remodeling proteins; in particular, those involved in endocytosis, organelle formation and maintenance, and cytokinesis in eukaryotic cells. Dynamins are molecular motors that modify membrane curvature (Fig. 1a). They are characterized by a GTPase domain, an elongated alpha-helical bundle that drives self-assembly, and the capacity to interact with lipids and membrane. They promote membrane tubules formation by self-assembling as an helical scaffold. GTP hydrolysis induces a conformational change in this scaffold that leads to further membrane constriction and possibly membrane fission or fusion [29]. In bacteria, multiple dynamin-like proteins (BDLP: Bacterial Dynamin Like Proteins) capable of modifying membrane curvature have been identified but their physiological roles are not entirely known. Some have been localized to the division septum, suggesting a role in cell division [30]. The two BDLP DynA and DynB for instance, are necessary for the cytokinesis event preceding sporulation in *Streptomyces venezuelae* [31]. Also, *Bacillus subtilis* DynA, a BDLP localized at the septa, mediates immunity against phage infection and membrane stress and it is also thought to be involved in membrane remodeling, since this protein is able to perform lipid mixing and membrane fusion in vitro, in a GTP-independent process [32–34]. A similar function in membrane repair and maintenance has been assigned to the *M. tuberculosis* BDLP IniA, which is capable to modify the membrane curvature of cardiolipin (CL)-containing liposomes and induce GTP-dependent membrane fission in vitro [35]. Finally, *Escherichia coli* LeoA, B and C are periplasmic BDLP implicated in outer membrane vesicles formation, although a direct interaction with lipids or a capacity to remodel membranes has not been demonstrated [36].

#### Bacterial cell division requires a coordination of membrane curvature modulating events

Another interesting illustration of physiological event requiring major membrane remodeling and membrane curvature modifications is bacterial cell division. Bacterial cells can be bounded by one or two membranes. Monodermic bacteria only possess one membrane, the cytoplasmic membrane, which is usually surrounded by a thick layer of peptidoglycan. In contrast, didermic bacteria have two membranes: the inner membrane (which corresponds to the cytoplasmic membrane of monodermic bacteria) and the outer membrane, separated by the so-called periplasm, which contains a peptidoglycan layer. During division, all those membrane layers must be remodeled to yield two independent bacteria. Membrane invagination required for bacterial cell division appears to be driven by forced membrane bending. Septum formation first requires the spatially regulated, hierarchical assembly of a multiprotein complex called the divisome. The correct positioning of the divisome at midcell is ensured by the Min and the nucleoid occlusion (NOc) systems, which are themselves dependent on and regulated by membrane lipids, especially anionic lipids microdomains found at the poles and septum [37, 38]. Amongst the more than 20 proteins that constitute the divisome, the tubulin-like GTPase FtsZ plays a central role in membrane invagination by oligomerizing into a dynamic, cytoplasmic Z-ring attached to the membrane via ZipA and FtsA. The complex further serves as a docking site for other divisome components. The role of FtsZ in membrane invagination has been debated. Its ability to display intrinsic curvature in its polymeric state and deform various artificial membranes in vitro [39], led to a model in which the cytoplasmic membrane is pulled inward by Z-ring constriction during cytokinesis. However, it has also been suggested that FtsZ only serves as a scaffold onto which the peptidoglycan remodeling machinery assembles. In the latter case, it is the growth of the septal cell wall that pushes the membrane toward the center of the cell [40]. In addition, the actin-like ATPase FtsA, which interacts with phospholipids via its C-terminus and bridges FtsZ to the membrane, was shown to induce membrane rearrangement in vitro and vesicle formation upon overexpression in *E. coli* [41]. It was thus proposed to facilitate membrane invagination by deforming the membrane at the septum site [41, 42]. Whatever its exact mechanism of formation, the membrane curvature generated upon membrane invagination in turn participates in the recruitment of negative curvature-specific proteins such as DivIVA, which further binds other players of cell division and localizes them at the septum site. The final steps of bacterial cell division (fusion and fission of the membrane(s) leading to the separation of the two daughter cells) are not characterized yet and it is still unclear whether specific fusion/fission proteins complexes are necessary or if membrane fission occurs spontaneously as a consequence of membrane curvature and/or protein crowding [43, 44]. A role for FtsA in this process has been proposed based on the occasional scission observed when FtsA was added to FtsZ-liposome in vitro [45]. However, this is inconsistent with the fact that FtsA, FtsZ and ZipA leave the septum before cell separation [40].

#### Orchestrated membrane curvature changes during sporulation

Sporulation is another event occurring in bacteria that involves extensive membrane remodeling. Under unfavorable conditions, the vegetative cells of some species of Gram-positive bacteria, such as *Bacillus* or *Clostridium*, produce resistant and metabolically dormant structures called spores. Sporulation starts with an asymmetrical cell division which generates a small cell (forespore) connected to the larger mother cell by two membranes separated by a thin peptidoglycan layer. This step requires the same divisome complex described for vegetative growth. However, whereas septum formation during vegetative growth avoids the nucleoid, during sporulation the septum closes over the chromosome, which is then translocated to the forespore. In *B. subtilis*, DNA translocation is performed by SpoIIIE, a homologue of *E. coli* FtsK which localizes at septal midpoint, possibly by its ability to sense regions of increased membrane curvature [46]. SpoIIIE is also required for membrane severing of the cytoplasmic bridge remaining between the mother cell and the forespore after DNA translocation. Although the exact mechanism by which SpoIIIE mediates this event is unknown, it was proposed that the protein forms multimeric channels in the mother cell and forespore membranes. Those channels finally assemble into an intramembrane trans-channel whose disassembly triggers membrane separation [47]. After severing of the membranes, the mother cell progressively engulfs the forespore, in a phagocytosis-like process, until the forespore is liberated in the cytoplasm of the mother cell, where it will further mature. During engulfment, the mother cell membrane generated at the division site expands and migrates on each side of the forespore until it totally surrounds it and the two leading edges reconnect next to the cell pole. The process of engulfment depends on two complementary ratchet-like mechanisms: the mother cell SpoIIDMP protein complex localized at the leading edge of the engulfing membrane, processively degrades the peptidoglycan synthesized ahead by the forespore and by doing so pulls the membrane forward [48, 49]. In addition, the forespore protein SpoIIQ interacts with the mother cell SpoIIAH in a zipper-like mechanism, which renders the membrane movement irreversible [50]. Once the two leading edges have meet, they fuse to liberate the forespore in the mother cell cytoplasm. In *Bacillus subtilis*, this final fusion event depends on SpoIIIE and FisB (Fission protein B) [47, 51]. FisB is a bitopic membrane protein with a large periplasmic region and a small cytoplasmic domain. Although the exact mechanism by which FisB triggers membrane fusion is still unknown, the protein is able to induce lipid mixing in vitro, in a process depending on the specific interaction between its periplasmic domain and CL. It is thus assumed that FisB, via this interaction, brings the two leading engulfment membranes in close contact before fusion [51]. As expected, sporulation is dependent on a reactivation of de novo membrane lipids synthesis [52]. CL for instance, is strongly enriched in the septum, the forespore and the mother cell engulfment membranes during sporulation [53] and accumulates in the mature spore [54]. Mutant strains producing only trace amounts of CL show delay in spore formation and produce reduced amounts of spores that are unable to germinate when placed back in favorable conditions [54]. CL enrichment might thus be important for the function of membrane proteins required for sporulation (e.g. FisB) or for their recruitment to specific regions of curvature. Membrane curvature-dependent localization has indeed been shown for *B. subtilis* SpoVM, which is necessary for spore maturation and localizes at the forespore surface by detecting positively curved membranes and inserting in them by an atypical amphipathic alpha-helix [55, 56].

### Evolutive origin of intracellular organelles

Although prokaryotic cells have been historically claimed as organelle-free organisms, several examples of intracellular membrane-restricted compartments have now been identified. For instance, intracellular membrane structures are naturally present in α-proteobacteria, an evolutive ancestor of γ-proteobacteria [57], where they either increase the efficiency of the cell bioenergetic metabolism (anaerobic anoxygenic photosynthesis, nitrifying and/or methanotrophic bacteria, etc.) or provide an evolutive advantage (magnetosome) [58].

#### Membrane curvature at the origin of mitochondria

The α-proteobacteria intracellular membrane structures have been proposed as potential ancestors of mitochondria inner membrane cristae after the discovery of a common membrane remodeling protein: alphaMic60 and Mic60 in α-proteobacteria and mitochondria, respectively [59]. The growth of intracellular membrane structures in both α-proteobacteria and mitochondria requires the assembly of the photosynthetic or respiratory protein complexes, which are known to induce strong membrane curvature [60–62]. Mic60 and its analogue alphaMic60 are part of the protein complex that presumably bends the membrane and stabilizes the *cristae* junctions in mitochondria (or inner membrane invagination points in α-proteobacteria). Although modern γ-proteobacteria lack the gene encoding alphaMic60 [63] and have lost the ability to physiologically produce inner membrane structures, heterologous overproduction of eukaryotic Mic60 restores the capacity of *E. coli* to produce those inner membrane structures [64]. This result implies that the ancestral mechanism for inner membrane proliferation of α-proteobacteria is not completely lost in γ-proteobacteria, but only *dormant*, and that it can be restored when certain conditions are met. The presence of CL in both prokaryotes and mitochondria is another argument often used to defend the endosymbiotic origin of mitochondria [65]. CL fulfills many biological roles in mitochondria and bacteria [66, 67]. Membrane curvature and CL seem to be linked, as CL depleted mutants show altered internal ultrastructure and function of mitochondria [68]. Furthermore, a recent study illustrates the importance of the formation of highly curved *cristae* for the correct accumulation of CL inside the mitochondria [69]. Notably, CL seems to be also related to physiological membrane remodeling processes in bacteria such as sporulation (“Influence of membrane curvature in membrane remodeling” section). Still, how CL, membrane curvature and membrane biosynthesis are exactly related remains a mystery.

#### Magnetosome formation

Magnetosomes are ~ 30–120 nm spherical, membrane-bound compartments that contain iron-rich magnetic particles. They organize as chains along the cell and allow magnetotactic bacteria to sense and orient in the geomagnetic field. They derive from the cytoplasmic membrane to which they may remain attached or not [70]. The mechanism of membrane invagination and vesicle formation, which seems to precede biomineralization, has not been completely deciphered but depends on the product of multiple *mam* (magnetosome membrane-associated) genes. Individual deletions of *mam* genes have identified four proteins involved in the biogenesis of the membrane of magnetosomes (MamI, MamL, MamQ and MamB) [71, 72]. However, the structure of those proteins has not been resolved and only speculations are available on the mechanism they use to bend the membrane and create the invaginations necessary for magnetosome formation [73, 74]. Besides, the overexpression of each one of those four genes alone is not sufficient to trigger membrane proliferation. More recent studies however suggest that at least some Mam protein might directly induce membrane curvature. In particular, MamY, a BAR domain-containing protein, interacts with liposome and induces liposome tubulation in vitro*.* Because a *mamY mutant of Magnetospirillum magneticum* showed altered magnetosome size-distribution, MamY was first proposed to be involved in membrane constriction [28]. However, MamY in vitro tubulation activity is specifically increased upon CL interaction, suggesting that MamY might recruit CL to the site of magnetosome formation to induce the formation of highly curved membranes [75]. Still, overexpression of MamY in *E. coli* or *M. magneticum* did not alter cell membrane morphology, confirming that in vivo, other factors are certainly needed to trigger membrane curvature and vesicle formation [28]. More recently, another study proposed that MamY represents a membrane positive curvature-sensing element and serves as a scaffold to properly align the chain of magnetosome parallel to the axis of the cell [76]. The role of CL in this function was however not tested.

#### Photosynthetic bacterial organelles

In some photosynthetic bacteria, intracytoplasmic vesicles called chromatophores contain pigments and light-harvesting proteins used to perform photosynthesis. Chromatophores function depend on the light-harvesting complexes 1 (LH1) and 2 (LH2) together with the reaction center (RC). These complexes, which are also directly implicated in chromatophore formation and shape determination, are thought to induce membrane curvature through a combination of wedging and scaffolding mechanism (Fig. 1). Indeed, the ability of these integral membrane proteins to bend and deform membranes depends on their capacity to oligomerize. The RC-LH1 complex, when monomeric, cannot bend membrane. However, RC-LH1 in complex with the small protein PufX forms dimers with the two monomers bent by a 146º angle [77–79]. In the absence of LH2, these dimers form tubular chromatophores in vivo [80]. LH2 is also sufficient to induce membrane curvature in *R. sphaeroides*. The protein forms hexagonally packed complexes, which are localized at high membrane curvature regions and, according to molecular dynamic simulation, could also induce membrane curvature [77, 81, 82]. The combined action of LH2 and RC-LH1-PufX would thus allow for the formation of spherical shaped chromatophores.

### Hijacking membrane remodeling: lessons learned from viral infection

**Fig. 2:**
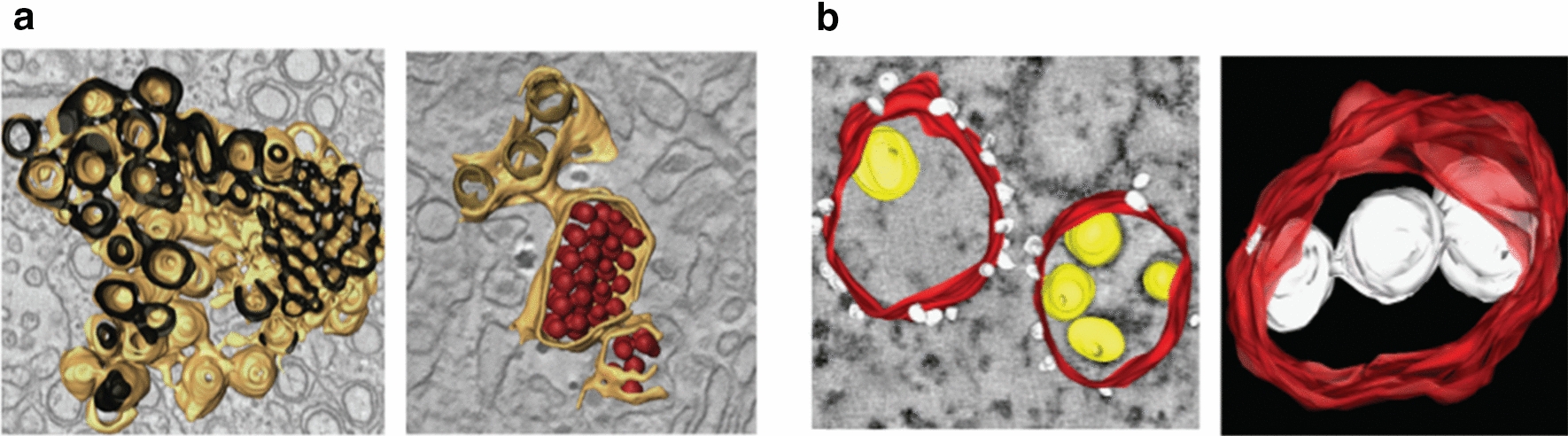
Electronic tomography reconstruction of the replication organelles of some + RNA viruses. **a** Left: Interconnected reticular network induced by dengue virus infection. The cytosolic face of the intracellular membranes is shown in brown and the ER lumen in black. Right: Viral particles (red) found in continuous ER cisternae. ER membranes are colored in light brown and inner vesicle membranes in dark brown [86]. **b** Left: Surface model of Kunjin virus replication organelles; ER membranes are colored in red, ribosomes in white and viral RNA in yellow. Right: Vesicles (white) connected to each other and to the ER membranes (red) [87]

In addition to the aforementioned membrane-remodeling physiological events, intracellular membranes can also be reshaped during infection by peculiar viruses able to usurp host lipid metabolism to create new compartments dedicated to their replication (replication organelles) (Fig. 2). Viruses infecting a large variety of hosts, ranging from bacteria and unicellular eukaryotes to vegetal and animal cells, have been described that trigger this phenomenon [83]. Among those, positive-sense single-stranded RNA viruses (+ RNA) infecting eukaryotic cells are the most studied. Because the + RNA of those viruses has the same sense as the cellular messenger RNA it is immediately translated when it reaches the cellular cytosol. Thus, viral proteins capable of modifying membrane curvature, which will be discussed in more detail in the following sections, are readily produced in the early infection stage [84, 85].

In addition, a correlation between membrane curvature and lipid biosynthesis during + RNA viral infection has been proposed [88]. Indeed, viral proteins modulating membrane curvature were also shown to promote the formation of membrane contact sites and the recruitment of host factors involved in lipid metabolism [89], in particular phosphatidylinositol-4-phosphate (PI4P) or phosphatidylethanolamine (PE) synthesis [90]. Interestingly, the accumulation of PIP4 or PE is often accompanied with an enrichment in sterol that might contribute to the stabilization of membrane curvature and is important for the replication of the virus [91].

In addition to + RNA viruses, other viruses containing double-stranded RNA (Reoviruses) as well as DNA viruses (Poxvirus, Vaccina virus, African swine fever virus, Frog Virus 3 and Paramecium Bursaria Chlorella Virus, giant Mimivirus Acanthamoeba polyphaga) also induce massive host membrane rearrangements. Although less studied, those viruses also rely on the production of proteins modifying the curvature of the host membrane [92, 93]. The membrane-enveloped double stranded RNA bacteriophages from the *cystoviridiae* family, such as phage φ6, are the only known enveloped phages and are evolutionarily related to the + RNA eukaryotic virus *picornavirus* [94]. They also produce proteins capable of bending the inner membrane of their hosts (Gram negative bacteria) which are necessary for virus replication [95].

In summary, certain viruses hijack the lipid metabolism of their hosts using specific proteins that modify membrane curvature and host co-factors to alter the lipid composition of the membrane to favor their own replication. However, how those factors are related to de novo membrane biosynthesis and viral replication organelles assembly remains elusive and should be further investigated.

### Inner membrane proliferation upon overproduction of some membrane proteins

**Fig. 3:**
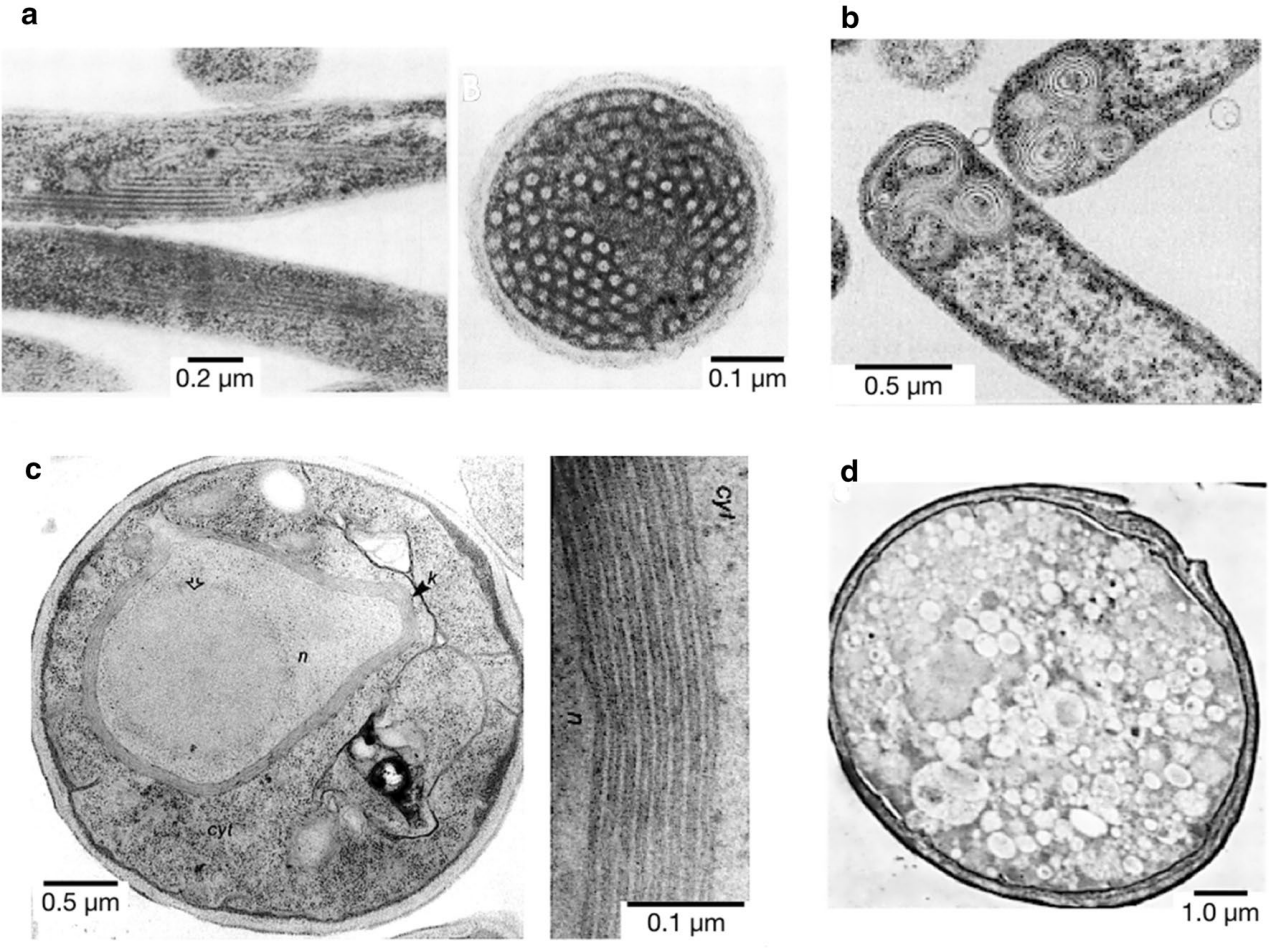
Negative-staining TEM pictures of some examples of inner membrane proliferation upon membrane protein overproduction. **a** Longitudinal (left) and transversal (right) sections of *E. coli* inner membrane tubules after fumarate reductase overproduction [96]. **b** Onion-like vesicles formed upon overproduction of protein 3A of Foot and Mouth Disease Virus (FMDV) [97]. **c** *S. cerevisiae* cell with the cytosol (cyt), nucleus (n) and the stacked membranes “*Karmellae*” (k) around the nucleus (n) (left) and detail of those membranous structures (right) after 3-Hydroxy-3-methylglutaryl-CoA reductase (Hmg-CoA) overproduction [98]. **d** Vesicles formed in *S. cerevisiae* upon overproduction of poliovirus protein 2BC [99]

Overproduction of recombinant membrane proteins is usually difficult due to various limitations, including a shortage of membrane space needed to accommodate the produced proteins. In a few peculiar cases however, overproduction of membrane proteins, either in prokaryotic or eukaryotic cells, has revealed an unexpected and intriguing ability of cells to synthesize an excess of internal membranes (inner membrane proliferation). In fact, these newly synthesized inner membranes often contain large amounts of well-folded recombinant proteins, holding great promises for biotechnological applications. Since the pioneer observation of Weiner et al. [96], only a few dozen membrane-associated proteins from prokaryotic (Table 1) and eukaryotic (Table 2) origin have been described that trigger non-physiological lipid membrane proliferation or *“inducible intracellular membranes”* (Fig. 3).

**Table 1:** Proteins triggering membrane proliferation in prokaryotes

Protein	Type	Origin	Expressed	Morphology	Protein arrangement	Lipid composition	Refs**.**
Fumarate Reductase	TM	*E. coli*	*E. coli*	Tubules	Helical (10 units per turn)	CL enrichment (~ 15% mol)	[96, 116]
Succinate Dehydrogenase	TM	*E. coli*	*E. coli*	Tubules and Vesicles	–	–	[117]
Mannitol Permease	TM	*E. coli*	*E. coli*	Vesicles	–	–	[190]
P6 (sp6.6 gene product)	TM	Phage PM2	*E. coli*	Vesicles	Unknown alone. Cage-forming protein associated with P3	–	[108]
P9 and P12	TM	Phage φ6	*P. syringae* *E. coli*	Vesicles	–	CL enrichment (~ 10% mol in *P.pseudoalcaligenes* infected by phage φ6)	[3, 112–114]
AlkB (alkane oxidation system)	TM	*P. oleovorans*	*E. coli*	Vesicles	–	CL enrichment (not quantified)	[191]
Tsr chemotaxis receptor	TM	*E. coli*	*E. coli*	Inner membrane invaginations	Crystalline pseudohexagonal (3 dimers of Tsr per repeating unit)	–	[125, 128]
*sn*-glycerol-3-P acyltransferase	TM	*E. coli*	*E. coli*	Tubules	Helical (6 dimers per turn)	No changes. Dependent on phage heat shock protein (PspA).	[120, 121, 192]
(b subunit of) F_0_F_1_ ATP synthase	TM	*E. coli*	*E. coli*	Tubules and vesicles	Unknown. Dimer formation	CL enrichment (~ 24% mol)	[6, 122, 123, 149]
P3A	M	FMDV	*E. coli*	Onion-like vesicles	Unknown. Possible oligomerization?	–	[97]
Caveolin-1	M	*H. sapiens*	*E. coli*	Vesicles	Well-defined supramolecular cage (160 monomers per cage)	PG and lysophospholipids enrichment (not quantified)	[104, 105]
MurG	M	*E. coli*	*E. coli*	Vesicles	–	CL enrichment (~ 22% mol)	[137]
LpxB	M	*E. coli* and *H. influenzae*	*E. coli*	Tubules and vesicles	–	PG and CL enrichment (not quantified)	[138]
PmtA	M	*A. tumefacensis*	*A. tumefacensis*	Vesicles	–	CL needed for membrane proliferation (not quantified)	[139]
alMGS	M	*A. laidlewii*	*E. coli*	Vesicles	–	PG and CL enrichment (not quantified)	[140–142, 193]

**Table 2:** Proteins triggering membrane proliferation in eukaryotes

Protein	Organism					
	Origin	Expressed	Morphology	Target organelle	Observations	Refs.
HMG-CoA	*S. cerevisiae* *S. pombe*	*S. cerevisiae*	“*karmellae”* stacked membranes around nucleus	ER	Soluble domain not required. Transmembrane helix alone not sufficient.	[98, 135, 150]
Cytochrome b5	*R. norvegicus*	*S. cerevisiae*	“*karmellae”* stacked membranes around nucleus	ER	Transmembrane domain disturbed by proline hinders membrane proliferation	[132]
Cytochrome P450	*C. maltosa*	*S. cerevisiae*	“*karmellae”* stacked membranes around nucleus + Tubules	ER	Minimum domain 1–33: contains hydrophobic helix and charged residues flanking it.	[129, 134, 220–222]
PMA2 (H^+^ ATPase)	*S. cerevisiae*	*S. cerevisiae*	Tubules	ER	–	[223]
RRp − 180 kDa	*C. lupus*	*S. cerevisiae*	“*karmellae”* stacked membranes around nucleus	ER	RBS not required for membrane proliferation. Increase of secretory pathway	[130, 224]
D_2S_ receptor	*H. sapiens*	*P. pastoris*	“*karmellae”* stacked membranes around nucleus	ER	–	[225]
sk2 Channel	*H. sapiens*	*P. pastoris*	“*karmellae”* stacked membranes around nucleus and ER	ER	–	[226]
B_2_ receptor	*H. sapiens*	*P. pastoris*	“*karmellae”* stacked membranes around nucleus	ER	–	[227]
LaminB receptor	*G. domesticus*	*S. cerevisiae*	“*karmellae”* stacked membranes around nucleus	ER	–	[228]
Pex12p	*S. cerevisiae*	*S. cerevisiae*	Multilayered membranes	Peroxisome	Observed morphology is dependent on expression level	[131]
Pex15p	*S. cerevisiae*	*S. cerevisiae*	Multilayered membranes	Peroxisome, ER	ER to peroxisome transport blocked	[210]
2BC	*Poliovirus*	*S. cerevisiae*	Vesicles	Vacuole	ER transport blocked	[99]
36 k protein IRV	Carnation Italian Ringspot Virus	*S. cerevisiae*	Vesicles	Mitochondria	–	[133]
Protein A	Flock House Virus	*S. cerevisiae*	Vesicles	Mitochondria	Retargeting of protein A to ER possible with ER specific sequence	[151]

All listed proteins are transmembrane proteins

From a morphological point of view, those inducible intracellular neo-membranes can be related to the bioenergetic compartments of α-proteobacteria and mitochondria [100], and/or replication organelles of + RNA viruses [88]. Furthermore, similarly to those “natural” intracellular compartments most of the proteins triggering *inducible intracellular membranes* (Tables 1, 2) also create zones with high membrane curvature [101]. For this reason, “natural” and “induced” intracellular membrane proliferation might share a more profound relation that goes beyond simple morphological resemblance.

## Mechanisms of protein-induced membrane curvature

Modulation of membrane curvature is often at the midst of both physiological and induced membrane remodeling processes. The induced membrane proliferation upon protein overproduction has, however, the advantage of being decoupled from the cell physiological regulations. For this reason, we will examine the current knowledge on the mechanism of inner membrane proliferation, focusing on the influence of membrane curvature not only on membrane morphology but also on phospholipid biosynthesis. In particular, four important questions about inner membrane proliferation upon protein overproduction remain: (1) how can overproduced proteins induce the deformation of the inner membrane creating different morphologies; (2) what are the characteristics of the proteins triggering lipid biosynthesis and, thus, inner membrane proliferation; (3) how are protein overproduction and de novo phospholipid biosynthesis coordinated; and (4) can we find regulatory mechanisms conserved across evolution explaining internal membrane proliferation in both prokaryotic and eukaryotic cells.

In order to yield the observed morphologies (vesicles, tubules, stacks of flat membranes, etc.) listed in Tables 1 and 2 and illustrated in Fig. 3, proteins inducing inner membrane proliferation must modify membrane curvature by means of one (or the combination of several) of the general mechanisms previously proposed (molecular motors, supramolecular scaffolding, asymmetric membrane interaction, wedging, see Fig. 1). However, the production of a pulling or pushing force will not be discussed in this section since none of the reviewed proteins is a molecular motor, nor a scaffolding protein interacting with any molecular motor.

### Protein–protein supramolecular interactions

The construction of a 3D supramolecular scaffold via supramolecular interactions is an efficient way of controlling cell membrane curvature and is used by many proteins involved in membrane remodeling processes (e.g. endocytosis, fission, motility, membrane trafficking, etc.) [102]. Most of the proteins inducing intracellular membrane proliferation (Tables 1 and 2) have been described to form supramolecular assemblies around the lipid bilayer. The membrane curvature and, consequently, the inner membrane morphology observed in electronic microscopy will depend on the shape, nature and concentration of monomers constituting the supramolecular scaffold.

#### Caveolin

Heterologously expressed caveolin-1 in *E. coli* cells is perhaps the best-characterized example of how a single membrane protein can shape the morphology of the newly synthesized lipid bilayer. Caveolin-1 is a scaffolding protein involved in the formation of vesicles (caveolae) arising from the plasma membrane of eukaryotic cells [103]. The formation of *heterologous-*caveolae (*h-*caveolae) derived from *E.coli* inner membrane is linked to the assembly of caveolin-1 into a supramolecular cage [104]. This cage contains around 160 caveolin-1 monomers and is similar in structure and size to eukaryotic caveolae. The three membrane-interacting domains and the oligomerization domain of caveolin-1 are required for inner membrane proliferation [105]. The formation of a regular and well-defined caveolin-1 scaffold seems to impose a strong local curvature on the cell membrane, causing the budding of vesicles coated with caveolin-1, and triggering the biosynthesis of phospholipids. As a consequence, monodisperse vesicles of the same size as those found in eukaryotic cells, accumulate in the *E.coli* cytosol.

#### Non-structural phage proteins

The overproduction of phage PM2 protein P6 represents another instance of membrane proliferation presumably induced by a supramolecular cage [106]. Although the structure of P6 has not been studied when it is overproduced in *E. coli* membrane, the structure of the entire PM2 phage has been determined by crystallography. This phage, which infects Gram negative bacteria from the *Pseudoalteromonas* genus [107], is composed of an icosahedral protein capsid containing a lipid membrane that encloses a double stranded DNA (dsDNA). P6 interacts with the viral lipid membrane but it is not a capsid forming protein. It associates with the P3 protein to form a well-ordered supramolecular structure that confers icosahedral symmetry to the lipid bilayer. P6 is inserted at the edges, whereas two P3 dimers stabilize the facets of the icosahedra. In other words, P6 is in charge of “welding” the lipid bilayer to create the icosahedral vertices. In the absence of P3, P6 is still able to impose a curvature to the *E.coli* inner cell membrane, producing vesicles [108]. Foot-and-mouth disease virus (FMDV) protein 3A is another example of a viral protein modulating membrane curvature. During the early stages of infection, FMDV, like other + RNA virus, remodels the ER membrane of its hosts (mammalian cells) to form the viral replication organelle, which provides a platform for viral RNA replication [109]. The interaction between the non-structural viral proteins (such as FMDV protein 3A) and the host phospholipids seems to be the trigger for host membrane remodeling [110]. When overexpressed in *E.coli*, FMDV protein 3A alone is able to deform the inner membrane, producing onion-like vesicles instead of its characteristic replication organelles [97]. The lack of other viral proteins or the differences in the nature and composition of phospholipids between eukaryotes and prokaryotes might explain this change in morphology. The precise mechanism of membrane deformation by FMDV protein 3A is unknown, but it requires the central amphipathic helix of the protein, together with the two cytosolic N- and C-terminal domains [111], which might interact with other viral proteins or induce oligomerization with other copies of FMDV 3A. Similarly, phage φ6 is a dsRNA bacteriophage, evolutionarily related to eukaryotic + RNA viruses (“Inner membrane proliferation upon overproduction of some membrane proteins” section), which infects Gram negative bacteria from the *Pseudomonas* genus. The overproduction of P9 and P12 phage φ6 proteins induce the proliferation of intracellular vesicles in *E. coli* and *P. syringae* [3, 112–114]. Both proteins (P9 and P12) are required to create the lipidic envelope of the phage [115]. P9 is a transmembrane protein, which is produced in large quantities in the early stages of infection and inserted in the inner membrane [11, 95, 112]. P12 is a non-structural protein and its role in the creation of the viral envelope is unknown, although it seems to somehow inhibit the degradation of P9 by the host proteolytic enzymes [112, 114].

#### Proteins involved in bioenergetic metabolism

Overexpression of some enzymes involved in energetic metabolism also induce membrane proliferation in *E. coli.* For example, fumarate reductase results in the formation of an array of densely packed lipid tubules in *E. coli* cytosol, that are severed from the inner membrane [116]. These lipid tubules are stabilized by a scaffold of fumarate reductase packed in a regular helical configuration containing 10 proteins per helix turn [96]. However, those tubules do not seem to fulfil any biological function, because the electronic transport chain is completely absent from the membrane expansions. Similar tubules are also observed when succinate dehydrogenase is overexpressed in *E.coli* [117]. Probably, the mechanism of tubule stabilization is similar to that observed with fumarate reductase due the structural and functional similarities between these two enzymes [118, 119]. Although the supramolecular packing of succinate dehydrogenase has not been studied in depth, the authors observed different morphologies (tubules or vesicles) depending on the expression level of the protein [117]. The supramolecular array of succinate dehydrogenase necessary to stabilize the tubule morphology might only be formed if the protein is produced at sufficient level. Thus, below a critical concentration, succinate dehydrogenase is still able to deform the membrane and yield vesicles but it is not capable of maintaining the tubule structure.

The *sn*-Glycerol-3-P acyltransferase is another example of protein inducing membrane tubules formation upon overproduction [120]. The individual molecules of *sn*-Glycerol-3-P acyltransferase are arranged in dumbbell-shaped dimers, which are packed in a left-handed helix along the tubule axis [121]. The association of six *sn*-Glycerol-3-P acyltransferase dimers completes a helix turn.

The whole F_o_F_1_ ATP synthase [122], and more efficiently, its b subunit alone [123], also produce vesicles and tubules detached from the inner membrane when overexpressed in *E. coli*. There is no data about the supramolecular packing of the b subunit of F_o_F_1_ ATP synthase in the lipid bilayer. Nevertheless, interactions between adjacent proteins seem to be important, since the removal of the cytosolic dimerization domain of the b subunit of F_o_F_1_ ATP synthase (residues from 53 to 122 [124]) inhibits tubules formation [123].

#### The serine chemotaxis receptor

The overproduction of the serine chemotaxis receptor (Tsr) from *E. coli* also triggers inner membrane proliferation [125]. Tsr is a transmembrane protein with a periplasmic domain that binds small molecules (Tsr is specific to serine) and a cytoplasmic domain associated with the adaptor protein CheW and the kinase ChewA [126]. In normal physiological conditions, cytoplasmic domains of adjacent Tsr form trimers of Tsr dimers, and self-assemble in two-dimensional clusters concentrated at the bacterial cell poles [127]. When overproduced, Tsr is also organized as trimeric assemblies of dimers [125]. However, because Tsr amounts are significantly increased, the two-dimensional clusters of Tsr can interact with each other creating a three-dimensional pseudo-hexagonal crystalline array that folds the inner membrane [125]. If this crystalline array is destroyed, e.g. by overproducing Tsr partners (ChewA and ChewW) at the same levels as Tsr, membrane proliferation is inhibited, even at high Tsr concentration in the membrane [128]. This result suggests that the high membrane curvature imposed by the crystalline array of Tsr is necessary to trigger phospholipid biosynthesis.

#### Examples in eukaryotic cells

Membrane curvature induction by protein overproduction is not restricted to prokaryotic hosts and has also been observed in eukaryotic cells (Table 2). Unfortunately, structural data on the arrangement of the recombinant proteins in the newly synthesized inner membranes are lacking. Still, there are some hints pointing to the presence of supramolecular scaffolds. For example, the minimal protein fragment of cytochrome P450 NADPH reductase and of canine ribosome receptor (RRp), which both induce membrane proliferation, include the charged residues flanking the hydrophobic transmembrane domain, as described for prokaryotic proteins [129, 130]. Moreover, it has been reported that, in some cases, the morphology of the newly produced membranes depends on the amount of overproduced membrane protein [131]. Disturbance of the 3D structure of the protein by introducing proline mutations [132], GFP fusions in critical positions [133], partially misfolding the proteins [134] or by deleting oligomerization domains [135], results in an altered morphology of the inner membrane structures. In addition, protein-protein supramolecular interactions are also required for inner membrane proliferation in mammalian cells [136]. The production of chimeras with cytochrome b5 transmembrane domains and a dimerization-prone GFP changes the morphology of proliferating membranes in the ER from stacks of membranes to a bi-continuous phase with cubic symmetry. Furthermore, membrane proliferation is also induced by fusing dimerization prone GFP to ER resident proteins; whereas each proteins overproduced separately, do not trigger membrane proliferation. Taken together, these features are in line with the observations made in prokaryotes, and suggest that protein-protein supramolecular interactions are a ubiquitous mechanism to control membrane curvature cells.

### Asymmetric interaction of proteins with only one leaflet of the lipid bilayer

Besides the formation of a supramolecular scaffold, the asymmetric insertion of a membrane protein into one leaflet of the lipidic bilayer can lead to a modification of the membrane curvature. Such a mechanism has been hypothesized for monotopic proteins inducing membrane proliferation such as MurG [137], LpxB [138] and PtmA [139] and confirmed by sequence comparison and in silico studies of alMGS [140, 141]. In addition, PtmA and alMGS have been purified and their ability to remodel synthetic liposomes into lipid tubules has also been demonstrated in vitro [139, 141, 142].

### Insertion of wedge-shaped proteins into the membrane

The insertion of wedge-shaped proteins into the lipid bilayer can also modulate membrane curvature [143]. Most transmembrane domains of membrane proteins introduce a packing mismatch in the lipid bilayer, which alters the membrane curvature. A well-known example of this mechanism is the wedge-shaped bacteriorhodopsin, a light-driven proton pump expressed in archaebacteria under anaerobic conditions. Bacteriorhodopsin is found in highly curved, specialized membrane microdomains (purple membranes), in which it forms trimeric, hexagonal units packed in a 2D crystalline lattice together with archaeal lipids [144, 145]. These lipids also contribute to the 2D crystalline packing as bacteriorhodopsin mutants with a constitutive wedge-shaped structure still need a specific lipidic environment to induce membrane curvature [146, 147]. Therefore, both phospholipid composition and protein tertiary 3D structure work together to modulate the membrane curvature in purple membranes.

It is remarkable how newly produced inner membranes of prokaryotes are enriched in cone-shaped non-bilayer forming lipids (CL or lyso-phospholipids) (Table 1). Similarly to wedge-shaped proteins, those cone-shaped phospholipids can also modulate membrane curvature. Their effect is expected to be less important than those induced by proteins, but not be negligible. Indeed, eukaryotic caveolae are enriched in phosphatidylinositol (PI) and cholesterol, which are important for the organization of lipid rafts. Similarly, *E. coli* adapts the phospholipid composition of *h-*caveolae replacing anionic PI by PG and non-bilayer forming cholesterol for lyso-phospholipids, only found in trace concentration in normal physiological conditions [104]. Symmetrically, in silico simulations show that membrane bending is facilitated by the incorporation in the lipid bilayer, of CL at levels similar to the ones observed in cases of membrane proliferation upon protein overproduction [148]. Furthermore, two examples have recently shown the importance of CL enrichment in the morphology of overproduced inner membranes. Firstly, *E. coli* mutants with reduced amounts of CL (2% vs. 24% mol in *wild type*) changed the organization of the produced inner membrane from organized tubules to onion like vesicles upon overproduction of the b subunit of F_o_F_1_ ATP synthase [149]. *E. coli* mutants completely depleted of CL confirmed this change of morphology and, in addition, were less efficient in triggering the proliferation of inner membranes [149]. Secondly, CL is necessary for the formation of inner membrane vesicles by PmtA, as it has been demonstrated both in vitro and in vivo [139]. In vitro, PmtA is unable to deform synthetic liposomes lacking CL and no vesicle-like structures where observed in vivo after PmtA overproduction in CL-deficient bacterial mutants. Together, both examples illustrate the central role of CL in the proliferation of inner membranes.

The phospholipid composition of the newly produced membranes in eukaryotes is largely unknown. To our knowledge, only a few indirect observations are available. For instance, “*Karmellae*” (or stacks of membranes surrounding the nucleus) produced by overproduction of 3-Hydroxy-3-methylglutaryl-CoA reductase (Hmg-CoA) seem to be enriched in neutral lipids, e.g. sterols [150]. However, no lipid quantification or identification was performed and this observation is only derived from a preferential staining with Nile Red, which has low specificity and indiscriminately stains all cell membranes. As previously discussed, the expression of some viral proteins, (2BC from poliovirus, 36K from Carnation Italian ringspot virus or protein A from Flock House virus) also induce different types of membrane in yeast (Table 2) [99, 133, 151]. In these cases, the creation of sterol-rich microdomains are required to assist membrane-remodeling proteins to effectively modulate membrane curvature [152, 153]. It is worth mentioning here the bacteriophage φ6, which induces a ninefold increase in CL levels in infected bacteria [115], reinforcing the importance of CL in intracellular membrane proliferation in prokaryotes.

### Summary and final considerations about protein-induced membrane curvature

Most of the proteins triggering membrane proliferation are able to locally modulate the membrane curvature. Independently of the precise mechanism involved in the creation of highly curved membrane microdomains, some common features are found in many of the reviewed proteins:

- The production of the membrane-interacting domains alone is not sufficient to modulate the membrane curvature. Additional protein domains are often required, suggesting that the induction of highly curved membrane microdomains is not a general consequence of membrane protein overproduction and insertion into the intracellular membrane.
- In some cases, the membrane curvature depends on the concentration of the overproduced proteins. A certain threshold of overproduced protein is necessary to trigger membrane proliferation. Different levels of protein concentration can lead to different membrane morphologies *in vivo.*
- The membrane curvature induced by proteins is often accompanied by a production of non-bilayer forming phospholipids (e.g. CL). These phospholipids can assist in the creation and stabilization of curved membrane domains and favor some membrane morphologies.

The membrane morphologies observed in vivo (vesicles, tubules, flat stacks of membranes, etc.) depend on the balance between protein 3D structure of the recombinant protein, its concentration, and the phospholipid membrane composition. Recently, Bonazzi et al. attempted for the first time to theoretically model the membrane morphology produced by arc-shaped proteins (or protein assemblies) modifying membrane curvature [154]. This theoretical model not only predicts the dependence of membrane morphology on protein concentration but also the existence of a vesicle-to-tubule transition, observed for some proteins listed in Tables 1 and 2. Furthermore, tubule-shaped membranes are formed independently of the shape of the protein when the concentration of proteins is high enough to cover more than 40% of the membrane surface area, which explains the prevalence of tubular membrane structures upon protein overexpression. This study strongly suggests that the mechanisms involved in membrane curvature induction and, as a consequence, the morphology observed upon protein overproduction, are predictable and determined by physical laws.

## Influence of membrane curvature on phospholipid biosynthesis in prokaryotic cell

The viability of bacteria directly depends on their ability to maintain membrane homeostasis and the electrochemical gradient in response to different environmental conditions. Lipid biosynthesis and modification are the most energy-intensive processes of membrane homeostasis, therefore it is not surprising that lipid metabolism is tightly regulated both transcriptionally and enzymatically [155]. Impressively, proteins discussed in this review have found a way to hijack this vital regulation, forcing bacteria to produce increased amounts of inner membranes.

**Fig. 4:**
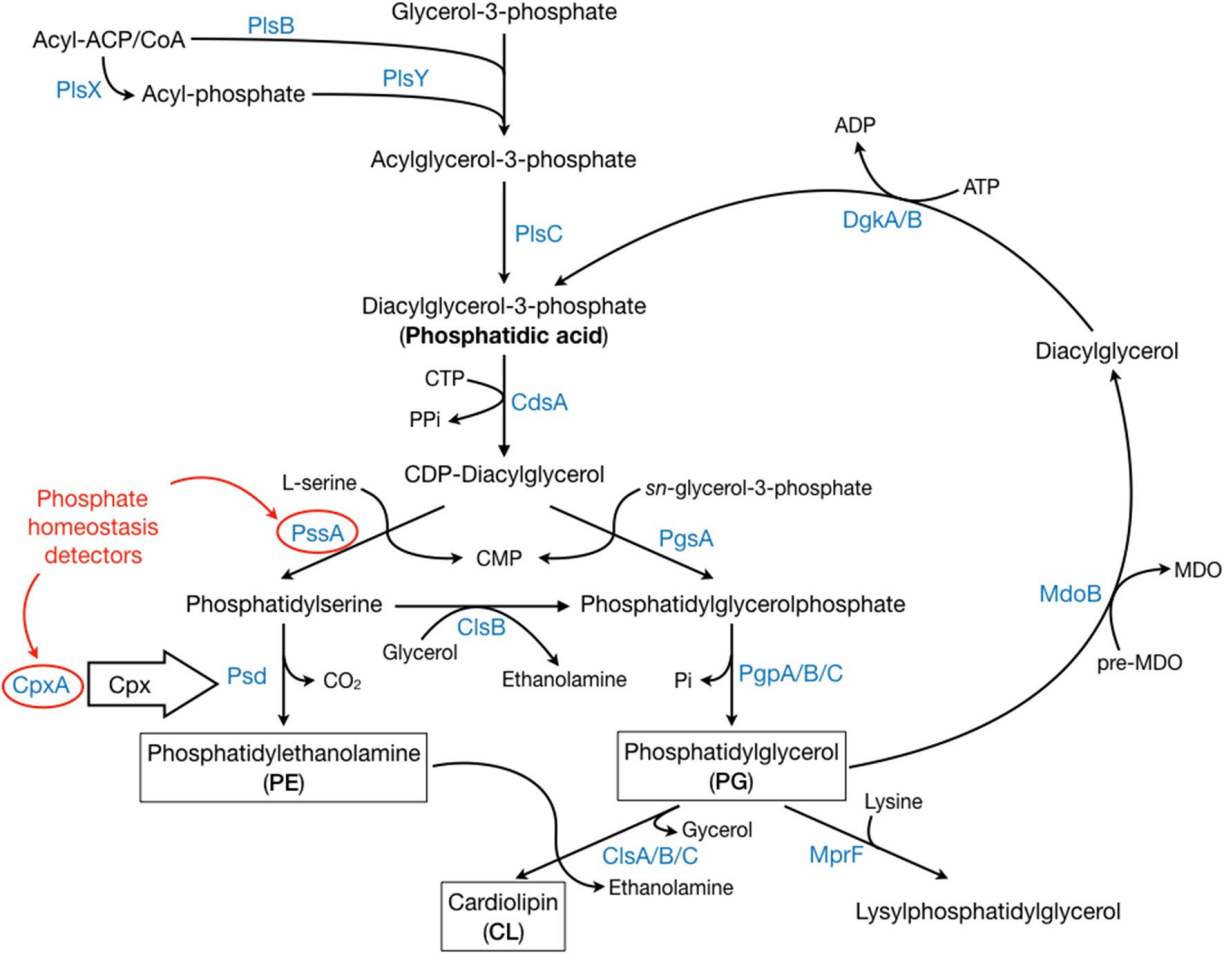
Brief overview of phospholipid polar head homeostasis in *E. coli*. Marked in red are the known membrane composition sensors. CTP: Cytosine TriPhosphate. CMP: Cytosine MonoPhosphate. MDO: Membrane Derived Oligosaccharides

Because fatty acid and phospholipid biosynthesis are coupled in *E. coli*, we will focus exclusively on the regulation of the biosynthesis of phospholipid polar heads, overlooking fatty acid metabolism [155]. Phospholipid metabolism and its regulation in prokaryotes (Fig. 4) have been extensively studied [156–158]. Still, even in the archetypical *E.coli*, many pieces are lacking to construct a complete vision of lipid metabolism. One of these missing pieces is how (membrane) protein expression is synchronized with lipid biosynthesis. Protein synthesis stopped after inhibition of lipid synthesis and re-started when lipid metabolism was restored [159–161]. Consequently, it appears that protein and lipid metabolisms are intimately connected and cross-regulated. However, the nature or mechanism of this regulation is unknown, even though some clues point to a regulation via multiple stress pathways [162–164].

### The importance of phospholipid homeostasis

*E. coli* maintains a constant ratio between zwitterionic phosphatidylethanolamine (PE), which accounts for about 75% wt. of total phospholipids, and anionic PG and CL, whose relative amounts depend on the physiological state (log- or stationary-phase) of the cells [155]. A feedback mechanism between the cross-regulated enzymes controlling the synthesis of PE and PG/CL (PssA and PgsA, respectively) maintains the homeostasis in phospholipid headgroup diversity (Fig. 4). PssA is a monotopic membrane protein that acts as a sensor, detecting changes in relative phospholipid composition (PE vs. PG/CL) in the lipid bilayer [165, 166]. It is active when associated with anionic phospholipids (PG and CL) and catalyzes the synthesis of PE. On the contrary, when anionic phospholipids become less available, PssA is deactivated, causing PgsA metabolic route to accelerate and to increase the synthesis of PG and CL.

Besides the aforementioned enzymatic regulation, phospholipid homeostasis is also subject to genetic control. Alterations in phospholipid composition stimulate the activation of several stress response pathways that ensure the maintenance of the bacterial envelope integrity (σ^E^, Cpx, Bae and Rcs) (Fig. 4) [167]. Even though all these regulation pathways are entangled, the Cpx system is of particular interest for phospholipid homeostasis in *E. coli* [164]. It activates the transcription of more than 100 genes, especially genes coding for inner membrane proteins and phospholipid metabolism, including Psd (an enzyme involved in the synthesis of PE, Fig. 4) [168–170]. Furthermore, the Cpx regulation pathway is controlled by CpxA, a transmembrane protein kinase located in the *E. coli* inner membrane, sensitive to modifications of the relative concentrations of anionic phospholipids in the lipid bilayer [171].

It seems clear that any alteration in the inner membrane phospholipid balance is compensated by the biosynthesis of the complementary type of phospholipids, either via enzymatic and/or genetic regulation. The enrichment in anionic phospholipids (especially CL) observed in most of the protein-triggered inner membrane proliferation (Table 1), most likely represents such an alteration. The constant biosynthesis of membrane proteins continuously alters phospholipid homeostasis and pushes *E. coli* to produce new phospholipids. The enrichment for specific phospholipids could be explained either by a selective interaction of the overproduced protein with the phospholipid type (PE or PG/CL), or by the creation of membrane microdomains with impaired accessibility to the homeostasis membrane sensors.

### Electrostatic interactions between anionic phospholipids and positively charged proteins

Overproduction of a membrane protein able to selectively bind anionic lipids, such as CL, could create a phospholipid imbalance in the inner cell membrane. Evidences supporting this hypothesis have been obtained in the case of the monotopic glucosyltransferase alMGS [140]. The alMGS domains that interact with phospholipids are enriched in positively charged amino acids [141], suggesting that they may selectively bind anionic phospholipid through electrostatic interactions. Indeed, the capacity of alMGS to alter phospholipid metabolism through its capacity to bind anionic phospholipids was later confirmed [142]. In addition, when overproduced, alMGS displaces the cyclopropane fatty acid synthase (CFA synthase) from its membrane binding site, inhibiting the synthesis of cyclopropanated fatty acids. Finally, phospholipid biosynthesis and alMGS overproduction are linked, as the supplementation of the culture media with lipid metabolism precursors leads to increased amounts of inner membranes with a concomitant increase in alMGS production [142]. Finally, alMGS overproduction also activates the σ^E^ and Cpx envelope stress responses, which can activate phospholipid metabolism as discussed in “The importance of phospholipid homeostasis” section. Besides alMGS, all of the monotopic membrane proteins listed in Table 1 (MurG, LpxB, caveolin-1 and PmtA) are known to bind to the lipid bilayer *via* electrostatic interactions with anionic phospholipids [104, 137–139]. Electrostatic interactions with transmembrane proteins have been less studied. Nevertheless, the activity of many respiratory complexes often depends on CL concentration [172]. Thus, it is not surprising that CL has been found to selectively interact with succinate dehydrogenase and F_o_F_1_ ATP-synthase [173, 174]. Indeed, it was recently demonstrated that the mitochondrial F_o_F_1_ ATP-synthase possesses a CL interaction site, conserved between yeast and bovine, which is enriched in positively charged amino acids [175, 176].

**Fig. 5:**
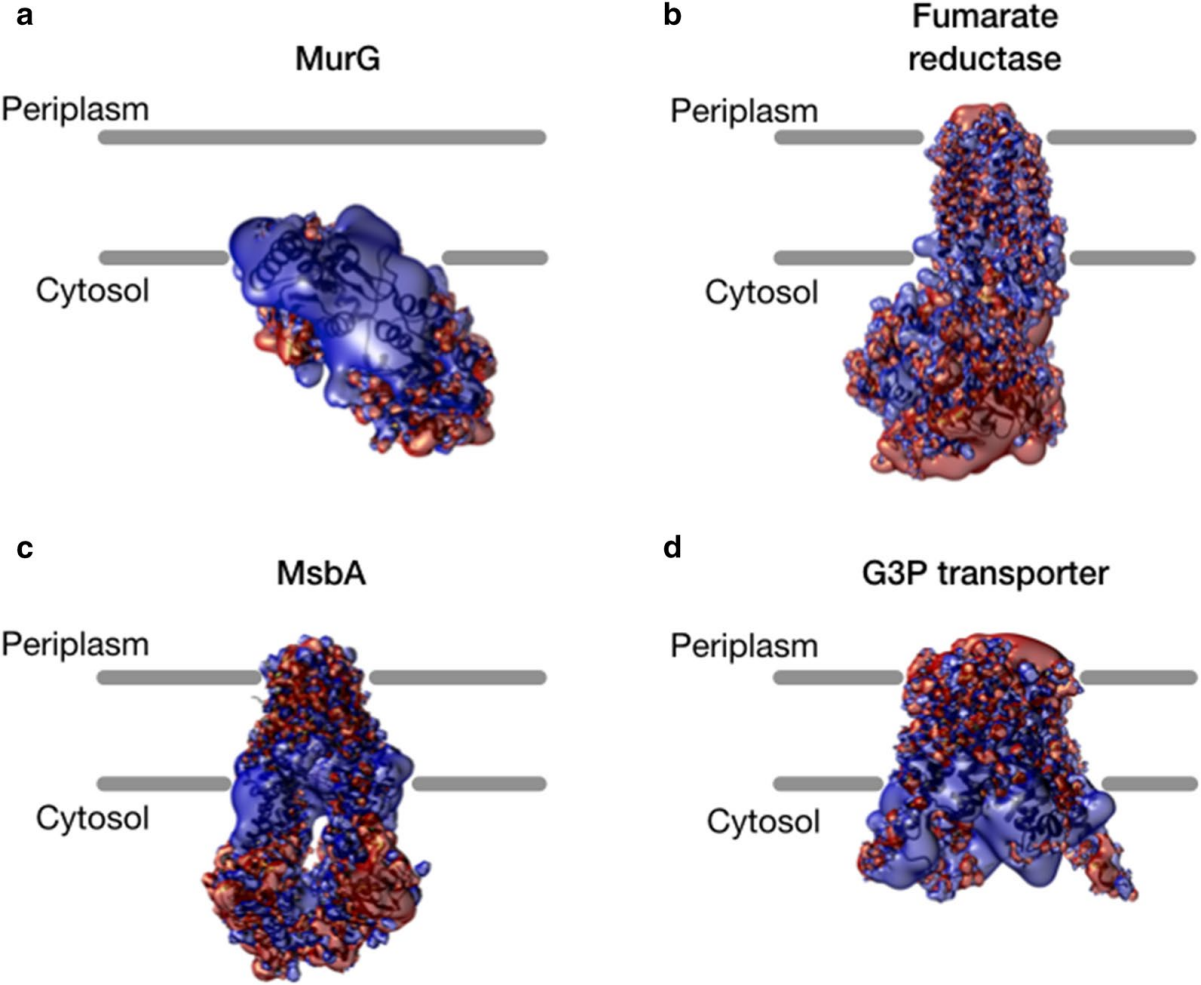
Isopotential electrostatic surfaces calculated for proteins available in the PDB. Charges were calculated at pH 7.5 with PDB2PQR server http://nbcr-222.ucsd.edu/pdb2pqr_2.0.0/ using Amber force field and naming schemes. In blue, positive isosurfaces at + 10 kT/e^−^ in red, negative isosurfaces at − 10 kT/e^−^. **a** Proteins triggering membrane proliferation when overproduced (monotopic MurG, PDB code 1F0K, [181] and transmembrane Fumarate reductase, PDB code 6AWF [119]); **b** proteins whose overproduction does not trigger membrane proliferation (MsbA, PDB code 6BPL [182, 183] and G3P transporter, PDB code 5XJ9 [184])

The presence of positively charged residues flanking transmembrane domains in the cytosolic leaflet of *E.coli* inner membrane is not, however, an exclusive feature of proteins triggering inner membrane proliferation. In fact, the “positive charges inside” rule [177], is a highly conserved mechanism to control the orientation of membrane proteins in the inner membrane [178]. This “positive charges inside” rule is conserved in most membrane proteins, not only in *E. coli*, but also in other prokaryotes and eukaryotes [179], while intracellular membrane proliferation has only been observed with a dozen of specific membrane proteins. To determine whether proteins able to induce membrane proliferation display specific charges distribution, we calculated the surface electrostatic potential of the proteins listed in Table 1 for which a structure is available in the PDB. Those proteins present a positive electrostatic lobe located nearby the phospholipid polar heads in the cytosolic leaflet of the inner membrane (Fig. 5a). However, this feature is far from being limited to the proteins inducing membrane proliferation. Indeed, MsbA and G3P transporter, two proteins that do not trigger intracellular membrane proliferation [180], also exhibit a marked positive electrostatic lobe near the cytosolic leaflet of the inner membrane (Fig. 5b). In conclusion, this positive electrostatic lobe is probably useful for membrane association and/or insertion in the correct orientation, but it is not sufficient *per se* to trigger inner membrane proliferation.

### Formation of CL microdomains

An alternative explanation for the perturbation of phospholipid homeostasis in a reduced accessibility of certain type of phospholipids to the membrane homeostasis sensors, that could be a consequence of lipid microdomains formation. Lipid rafts enriched in sterols, sphingolipid and some proteins are a well-established membrane organization element in eukaryotic membranes [185]. The existence of membrane lipid microdomains, analogous to the lipid rafts of eukaryotes, has also been reported in prokaryotes [186]. These membrane microdomains associate with specific membrane protein complexes and are enriched in particular lipids (notably CL) [66, 158]. Moreover, the spontaneous breaking of membrane symmetry with subsequent enrichment in CL in areas of high negative curvature has been theoretically modeled and also experimentally observed in *E. coli* membranes [148, 187, 188]. Very recently, this CL clustering and enrichment of highly curved membrane areas has been experimentally quantified [189]. It should be noted that in both theoretical and experimental designs an external force is necessary to impose high membrane curvature, as a mere accumulation of CL alone is not sufficient to curve the membrane enough to induce further CL clustering.

**Fig. 6:**
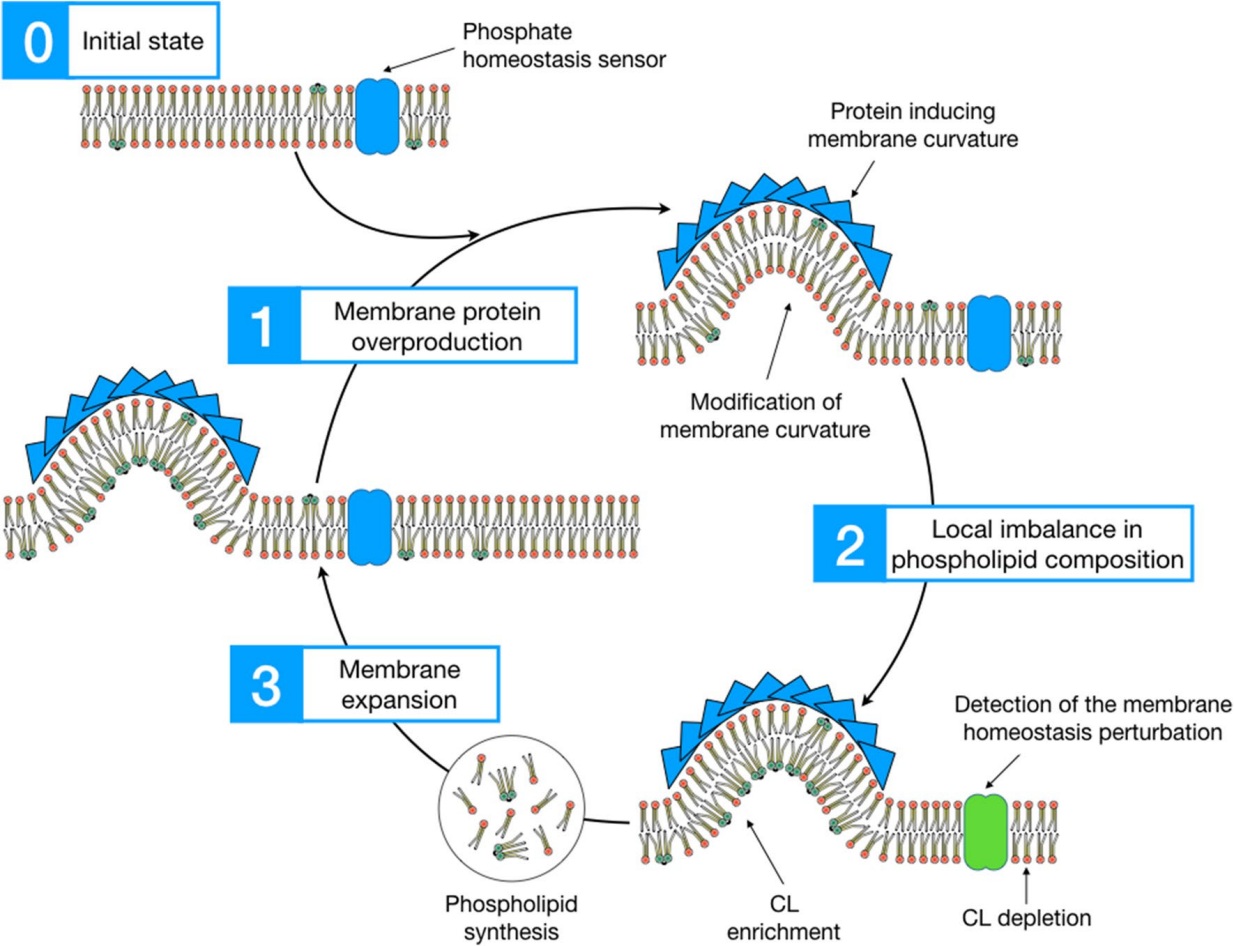
Proposed mechanism for phospholipid biosynthesis triggered by membrane protein overproduction. TM: Transmembrane protein; M: monotopic protein; FMDV : Foot and Mouth Disease Virus

In this regard, the insertion of a membrane protein (or supramolecular complex of proteins) inducing a high local curvature would be needed to provide the necessary force to bend the membrane and induce CL clustering. This could in turn lead to an anionic phospholipid depletion in the non-curved zones of the membrane, which will subsequently be detected by the phospholipid homeostasis sensors as a signal to start lipid biosynthesis. Then, the newly synthesized lipid membranes would allow for more membrane protein insertion, thus, closing the cycle (Fig. 6). As previously discussed in “Mechanisms of protein-induced membrane curvature” section, all the proteins triggering inner membrane proliferation have the capacity to modify the membrane curvature and, hence, to induce CL clustering. Of note, this hypothesis can explain not only the production of inner membranes but also the enrichment in CL (instead of anionic PG that would be expected in the case of the electrostatic interactions hypothesis) observed in most examples of intracellular membrane proliferation upon protein overproduction. Furthermore, it could be the missing link to the ancestral mechanism of inner membrane compartments observed in α-proteobacteria and mitochondria, where both the insertion of membrane curvature-inducing protein complexes and CL clustering are required.

## Influence of membrane curvature on phospholipid biosynthesis in eukaryotic cells

Unicellular eukaryotes (especially yeasts) are, like prokaryotes, well-known hosts used for protein production and biotechnological applications. As in prokaryotes, intracellular membrane proliferation can also be induced by some membrane proteins overexpression in unicellular eukaryotes (mostly documented in *S. cerevisiae* and *P. pastoris*). To date, only transmembrane (not monotopic) proteins have been reported to trigger membrane proliferation in yeasts (Table 2). The first reports of membrane proliferation in eukaryotes described nuclear membrane structures (*Karmellae*), but since then, some have been shown to also originate from other cell organelles such as the endoplasmatic reticulum (ER), mitochondria, peroxisome or vacuole (Table 2). Membrane proliferation can now be specifically targeted to particular cellular organelles by engineering chimera proteins containing *ad hoc* signal peptides [151].

### The unfolded protein response

**Fig. 7:**
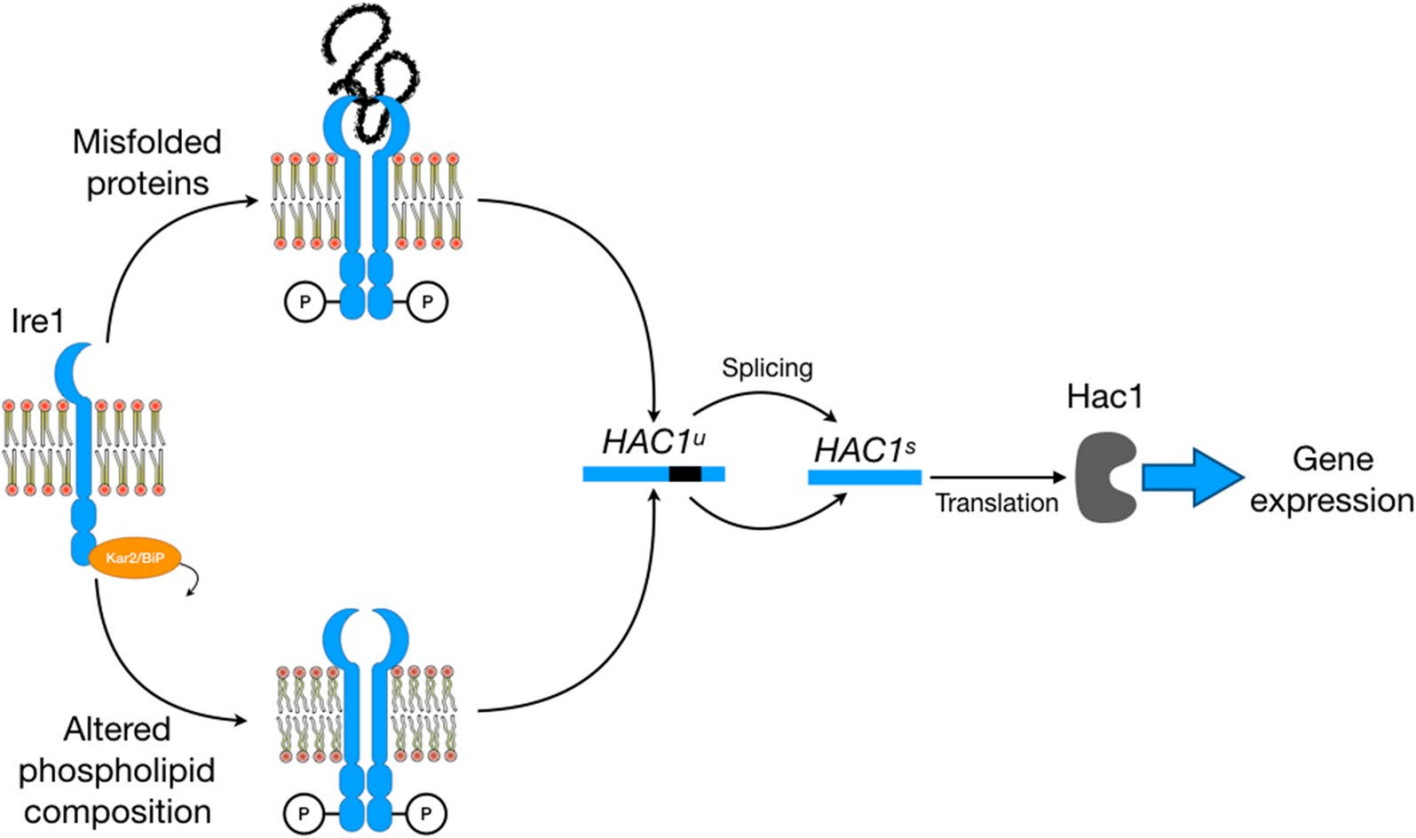
UPR phospholipid sensor. Ire1 is able to detect the presence of misfolded proteins or alterations of phospholipid composition in the membrane of the ER and is activated by dimerization and autophosphorylation. This activation leads to an unconventional splicing of *HAC1* mRNA and the translation of Hac1, which activates gene expression and phospholipid biosynthesis

The relationship between expression levels of recombinant membrane proteins and phospholipid biosynthesis has been studied more in depth in eukaryotes than in prokaryotes. Early reports on the activation of the inositol response pathway and the Unfolded Protein Response (UPR) in *S.cerevisiae* overproducing Hmg1 were the first attempts to link membrane proliferation to protein overproduction [194]. In yeast, UPR is controlled by Ire1, an integral ER membrane protein with an ER luminal domain sensitive to the presence of misfolded proteins (via the release of the chaperone Kar2, also known as BiP) and a transmembrane and proximal domain sensing alterations of lipid composition in the ER membrane [195, 196]. Either an accumulation of unfolded proteins or an imbalance of lipid homeostasis constitute life-threatening events, which are tightly controlled by Ire1 (Fig. 7) [197]. Activation of Ire1 triggers the production of Hac1, a transcription factor that controls ca. 381 genes, including those of phospholipid biosynthesis, ER-associated protein degradation, protein translocation across ER membrane, vesicular trafficking, cell wall biogenesis, and vacuolar protein sorting [198]. It was then postulated that the overproduction of a membrane protein is a perturbation that could activate UPR response and, indirectly phospholipid biosynthesis through Ire1 sensor. Ire1 knock-out yeasts (*S. cerevisiae* ∆ire1) were constructed to test this hypothesis. However, membrane proliferation upon overproduction of P450 cytochrome or Pex15p was not impeded in ∆ire1 yeasts [199, 200]. Interestingly, ∆ire1 yeasts were still able to produce Kar2/BiP, which is a part of the UPR response. Thus, the authors postulated an alternative (and unknown) Ire1-independent mechanism for UPR activation. Later, these observations were expanded to HMG-CoA reductase isozyme (Hmg1) overproduced in ∆ire1 yeasts. In this case, membrane proliferation was achieved in complete independence from Ire1 and secretion of Kar2/Bip chaperone. Consequently, the authors concluded that, at least for Hmg1, membrane proliferation phenomena should be unrelated to UPR. However, the activation of UPR pathway seems somewhat advantageous for inner membrane proliferation, as the overproduction of the transcription factor Hac1 (the main product of UPR), using an external expression plasmid improved the production yield of membrane proteins and intracellular membranes [201, 202]. Interestingly, overproduction of Hac1 alone changes the morphology of the ER membrane to a cubic phase and increases the Kar2/BiP chaperon levels [201]. As previously discussed, bi-continuous cubic phase ER is often observed during the infection by + RNA viruses [89], which are known to induce membrane proliferation in both prokaryotes and eukaryotes. Additionally, stacks of *Karmellae*-like membranes surrounding the yeast nucleus are produced upon coexpression of Hac1 with a membrane protein, which alone would not trigger membrane proliferation [202].

### Inositol regulation pathway and the importance of phosphatidic acid

**Fig. 8:**
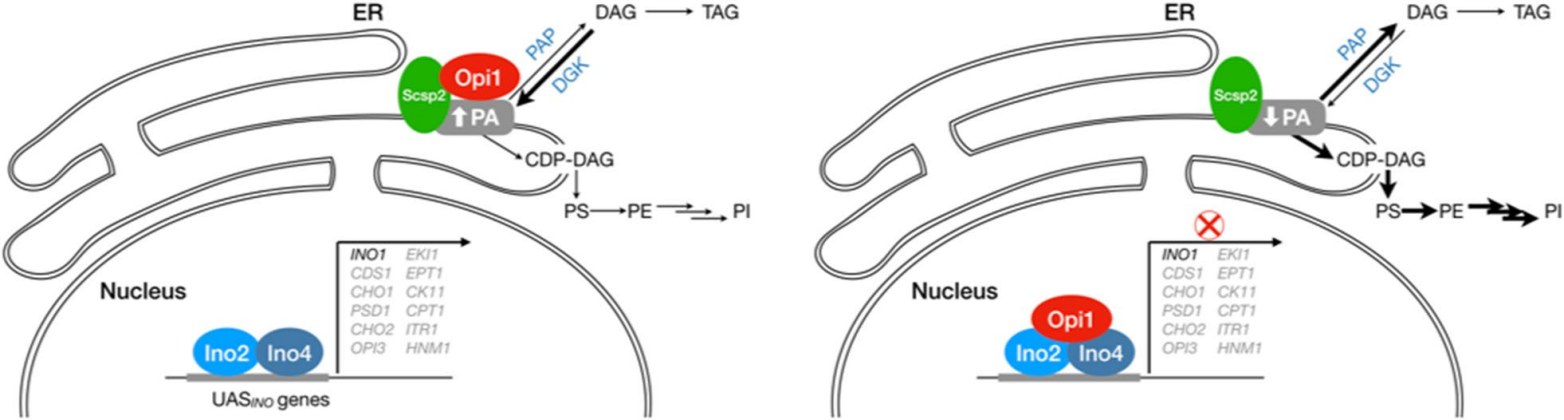
Inositol dependent phospholipid sensor. When PA levels are high (i.e. low inositol concentration), Opi1 interacts with Scsp2 and is sequestrated in the ER membrane (left) and *INO1* and other inositol-sensitive genes are expressed. When the PA levels drops down, Opi1 detaches from Scsp2 and migrates to the nucleus where it represses the expression of inositol-sensitive genes (right)

How to reconciliate the fact that membrane proliferation in yeasts is at the same time influenced by and independent of UPR? A plausible explanation is the existence of redundant sensors capable of activating, independently of UPR, the biosynthesis of phospholipids. PI and its precursor, inositol, are implicated in the inositol regulation pathway (*INO1* genes), which is activated during membrane proliferation after canine RRb production in yeast cells [203]. *INO1* promotes the synthesis of phospholipids (and many other inositol-sensitive genes) and is regulated independently of UPR by the couple of activators Ino2/Ino4 and repressed by Opi1 (Fig. 8) [204]. This regulation system is clearly related to membrane proliferation, as mutants deficient of Ino2 failed to produce intracellular membranes upon RRb expression, whereas deletion of the repressor Opi1 favors the proliferation of inner membranes [203]. All the genes under inositol regulation (including *INO1*) are, in fact, controlled by phosphatidic acid (PA) levels, which is a central regulator of both the synthesis of phospholipids and reserve lipids (triacylglycerides) in *S. cerevisiae* [205]. Opi1 is a soluble protein associated to a transmembrane protein (Scs2), which acts as a membrane sensor for PA microdomains [206, 207]. When PA concentration is sufficient, Opi1 remains inactive and anchored to Scs2 in the ER membrane. On the contrary, when PA concentration drops down, Opi1 is released and imported to the nucleus where it represses the phospholipid synthesis.

### Other regulatory mechanisms

In addition, the regulation of phospholipid homeostasis in *S. cerevisiae* is controlled by multiple biochemical and genetic factors and goes well beyond UPR and inositol regulation pathways [208]. Therefore, the existence of additional regulatory pathways to control phospholipid biosynthesis upon protein overproduction cannot be ruled out. In fact, intracellular membrane proliferation of ER in *S. cerevisiae* is associated with altered membrane trafficking. For example, overproduction of Sec12p blocks the ER-to-Golgi intracellular trafficking of *S.cerevisiae* and induces the formation of clusters of the chaperone Kar2/BiP, like in UPR pathway [209]. Similar blockage of intracellular trafficking was also observed after overexpression of the poliovirus 2BC protein and the peroxisomal Pex15p protein, which both accumulate newly formed ER membranes [99, 210]. Conversely, the overproduction of the canine RRp enhances the secretory pathways in *S. cerevisiae* [130]. Of note, this altered intracellular trafficking is not a general scenario for all the protein-induced intracellular membrane proliferation in eukaryotes, as exemplified with Hmg1 [209]. It is not clear whether these alterations of cellular trafficking are only a consequence of membrane proliferation or, on the contrary, contribute to inner membrane proliferation in eukaryotes. In any case, regardless of the precise mechanism, the biosynthesis of phospholipids also seems to be controlled by the membrane composition in eukaryotes.

### Cardiolipin and phosphatidic acid membrane microdomains: a universal regulator mechanism for phospholipid biosynthesis conserved through evolution?

PA seems to be a central actor in phospholipid biosynthesis regulation in eukaryotes [208]. For instance, increased PA cellular levels, either by a lack of PA degradation due to lower PA phosphatase activity or by an increase in PA concentration due to an overproduction of diacylglycerol kinases, leads to an expansion of the nuclear membrane [211–213]. It should be noted that this membrane expansion in the absence of any membrane protein modulating membrane curvature leads to yeasts with an aberrantly large nucleus, without any organized membrane morphology (e.g. stacks of membranes, tubules or vesicles). Furthermore, PA and CL also play a central role in the regulation of membrane dynamics (fusion and division) in mitochondria [214].

**Fig. 9:**
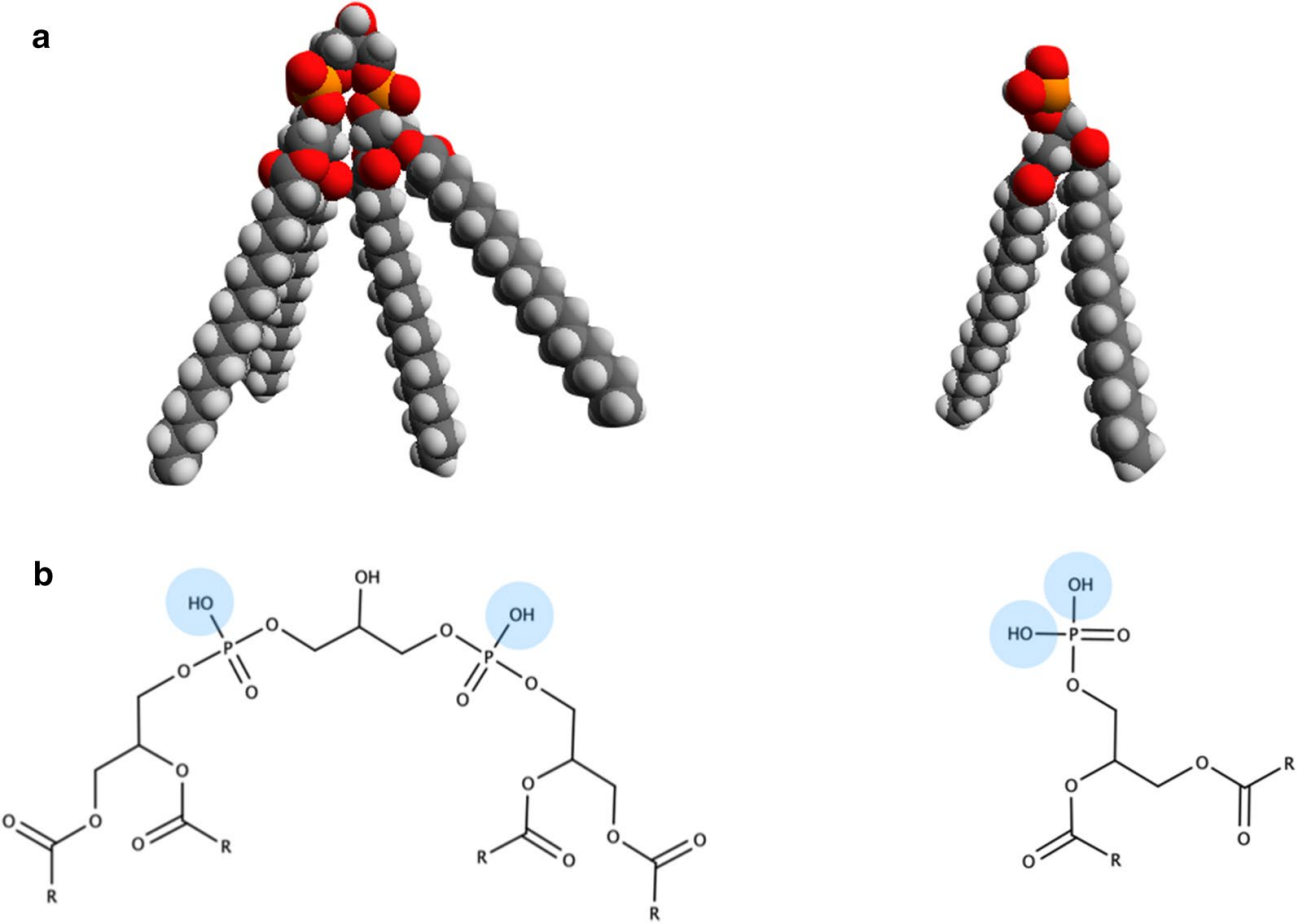
**a** 3D models of fully extended CL (left) and PA (right) showing the cone-shaped nature of these phospholipids. **b** The two ionizable positions have been highlighted in blue in the 2D chemical representation

From a physicochemical point of view, the phospholipids found at the core of membrane proliferation in eukaryotes and prokaryotes (PA and CL), are strikingly similar (Fig. 9) [215]. Both PA and CL are cone-shaped anionic phospholipids that accumulate in zones of negative curvature in biological membranes. Furthermore, both of them have two ionizable positions (Fig. 9), determined by two distinct pK_a_ values [216, 217]. Their exact values and hence, the ionization state of PA and CL, depend on the membrane local environment. Indeed, different ionization states of PA controlled by lipid-protein interactions have been proposed to explain the regulatory role of PA in eukaryotes [216]. Similarly, CL undergoes changes in lipid packing as a function of its environment, external pH and divalent cations [218, 219].

As discussed before, proteins inducing membrane proliferation in both prokaryotic and eukaryotic cells are characterized by their ability to bend the membrane. At the same time, cone-shaped anionic PA and CL share a marked preference for negatively curved regions of the membrane. Therefore, the disturbance of phospholipid homeostasis via the creation of membrane microdomains enriched in PA or CL might be a central regulator of phospholipid metabolism conserved through evolution.

## Conclusions and perspectives

Despite the variety of inducible membranes and host cells reported in the literature, the proteins triggering membrane proliferation share some common properties. They are able to modify membrane curvature and hijack the regulation of phospholipid synthesis, where anionic, non-bilayer-forming phospholipids (CL and PA) seem to play a central role. We reviewed the mechanisms involved in phospholipid homeostasis, identifying their possible coordination with membrane protein biosynthesis. The hypothesis that phospholipid production might be induced by electrostatic interactions between anionic phospholipids and a positively charged lobe of the protein has been ruled out. Taking into account all the reviewed information, we propose a general mechanism of intracellular membrane proliferation in which the overproduced recombinant proteins can induce high curvature local areas, creating clusters of anionic, cone-shaped phospholipids (CL in prokaryotes and PA in eukaryotes). The accumulation of these phospholipids in the curved microdomains causes a phospholipid imbalance in the non-curved areas of the cell membrane. This local imbalance is likely to be detected by the phosphate homeostasis sensors, which in turn, will stimulate phospholipid biosynthesis. Upon the continuous accumulation of the recombinant protein (or their supramolecular assemblies), new curved microdomains are produced, and the cell is forced to constantly synthesize new phospholipids to maintain phospholipid homeostasis. The final consequence is an expansion of the intracellular membranes displaying the different morphologies observed in cellulo. This general mechanism of intracellular membrane proliferation shares common features with the formation of energetic compartments in α-proteobacteria (and mitochondria), and for some viruses, which remodel the inner membrane structures of the host to create their replication organelles. In both cases, the membrane expansion is triggered by the insertion of proteins that modulate membrane curvature, causing the accumulation of non-bilayer forming lipids (CL or PA). In summary, the modification of membrane curvature can act *in cellulo* as an inducer of phospholipid biosynthesis and membrane expansion and it is probably a conserved feature through evolution.

## Data Availability

Not applicable.

## Abbreviations

alMGS: monoglucosyldiacylglycerol synthase
alphaMic60: α-proteobacteria Mic60 analogue
BAR: Bin Amhiphysin Rvs
BDLP: bacterial dynamin like proteins
B_2_ receptor: Human Bradykinin subtype 2 receptor
CL: cardiolipin
CMP: cytosine monophosphate
CTP: cytosine triphosphate
DivIVA: cell division protein IVA
D_2S_ receptor: dopamine receptor type 2s
ENTH: Epsin NH_2_ -Terminal Homology
FisB: fission protein B
FMDV: foot and mouth disease virus
FtsA/K/Z: filamentous temperature sensitive protein A/K/Z
Kar2: endoplasmic reticulum chaperone BiP
G3P: glycerol-3-phosphate
Hac1: homologous to Atf/Creb1
Hmg-CoA: 3-hydroxy-3- methylglutaryl coenzyme A
Hmg1: isozyme 1 of Hmg-CoA
*INO*: inositol regulated genes
Ire1: serine/threonine-protein kinase/endoribonuclease
LH: light harvesting complex
LpxB: lipid-A-disaccharide synthase
*mam*: magnetosome membrane-associated
MDO: membrane derived oligosaccharides
Mic60: subunit Mic60 of mitochondrial contact site and cristae organizing system complex
MurG: UDP-*N*-acetylglucosamine–*N*-acetylmuramyl-(pentapeptide) pyrophosphoryl-undecaprenol N-acetylglucosamine transferase
MsbA: lipid A export ATP-binding/permease protein MsbA
NOc: nucleoid occlusion
Opi1: transcriptional repressor OPI1
PA: phosphatidic acid
PE: phosphatidylethanolamine
Pex12p: peroxisome assembly protein 12
Pex15p: peroxisomal membrane protein 15
PG: phosphatidylglycerol
PI: phosphatidylinositol
PI4P: phosphatidylinositol-4-phosphate
PMA2: plasma membrane H+-ATPase 2
PmtA: phospholipid *N*-methyltransferase A
PgsA: CDP-diacylglycerol-glycerol-3-phosphate 3-phosphatidyltransferase
PssA: phosphatidylserine synthase
PufX: intrinsic membrane protein PufX
RC: reaction center
+RNA: positive-sense single stranded RNA
RRp: canine ribosome receptor
Scs2: membrane protein-associated SCS2
Sec12p: guanine nucleotide-exchange factor SEC12
Sk2 channel: small conductance Ca2+-activated K + channel
SpoIIDMP: stage II sporulation proteins D, M and P complex
SpoIIIE: stage III sporulation protein E
SpoVM: stage V sporulation protein M
Tsr: serine chemotaxis receptor
UPR: unfolded protein response
ZipA: cell division protein ZipA
